# Hospital and Institutional News

**Published:** 1917-05-19

**Authors:** 


### HOSPITAL AND INSTITUTIONAL NEWS.

### A NEW DIRECTOR-GENERAL OF THE NAVY MEDICAL SERVICE.

It is officially announced that on June 1, 1917, Surgeon-General W. H. Norman, C.B., will succeed Surgeon-General Sir Arthur May, K.C.B., F.R.C.S., K.H.P., as Director-General of the Medical Department of the Royal Navy, and that he has been granted the relative rank of Vice-Admiral from that date. Sir Arthur May, the retiring Director-General, succeeded Sir James Porter in the office which he has held with so much credit; and to these two officers is due very largely the excellent state of health which has obtained amongst the *personnel* of the Navy since the outbreak of the war. Contrary to all expectations formed beforehand, the casualties amongst naval officers and men have not been, comparatively, severe; so that there has been little opportunity for testing, on a scale comparable with that afforded by the operations of the Army, the perfection of the arrangements dealing with the wounded from naval engagements. Even the Jutland fight produced casualties quite moderate in dimensions. There is every reason to believe, however, that Sir Arthur May's dispositions are capable of meeting any emergency; and he will carry with him in his retirement the good wishes of those who have had the fortune to serve under him.

### THE WAYS OF THE WAR OFFICE.

A VERY strong line is taken, and on the face of things quite properly, in a letter to the *Times* written by Dr. Howard Marshall, of Cirencester, about the way in which the War Office has treated the medical officers of auxiliary hospitals. At the outbreak of war, as he remarks, many civilian medical officers placed their services gratuitously at the disposal of voluntary aid hospitals for military patients. Great though the tax on their time and energies has been, many such doctors have continued right down to the present day to serve the State in this way without reward, and sometimes to the detriment of their private practices. It has been discovered, however, that as early as June 1915, the War Office authorised the payment of these medical officers on a certain scale, which, however adequate or inadequate it may be, is at least some recognition of services rendered to the wounded. "This decision," says Dr. Marshall, and our information leads us to believe that he may be right, "was carefully concealed from the civilian medical officers by official instructions, while it was carefully laid down that the payment . . . could only begin from the date of application," and was not to be retrospective. Moreover, payment was to be contingent on the signing of a form which the War Office refused to supply. There is more of Dr. Marshall's indictment, but the gist of it is given above, and it is to be hoped that the War Office can advance some explanation and defence of what appears to be as mean and niggardly a transaction as ever disgraced a British Government office. Failing some reasonable explanation, or a retractation of the objectionable features of the action of the War Office, we hope some member of the House of Commons will take the matter up at question time. The ways of the War Office always have been beyond the comprehension of ordinary straightforward, common-sense citizens, and this is a typical instance of their crooked methods and tortuous policies, especially stupid in that it was bound to be exposed sooner or later.

### A CURIOUS ANNOUNCEMENT.

The following announcement, which we quote from a provincial newspaper, is worth quoting: "Lady Somerleyton has been obliged to close her Primary Military Hospital near Newmarket, of which she has been matron for two years and nine months, owing to the deficiency of doctors." It will not be the first private institution which has had to be closed, but it is curious to read of a "Primary Military Hospital" with which Lord Somerleyton is so closely associated, that has had to close owing to a shortage of doctors.

### AN EXPERT'S ENCOURAGEMENT.

The Hon. Arthur Stanley is indefatigable in his public work for the Red Cross and the nurses, and he lately made a remarkable speech at Liverpool in inaugurating a new division. One of the few satisfactory results of the war had been, he said, the fusion of the Order of St. John with the British Red Cross Society. Another improvement had been brought about in respect of the treatment of the wounded in the East, and he was assured that not even in France were they better treated than at Bagdad at present. He paid a special tribute to the voluntary hospitals in France, and praised the design and management of the Liverpool Merchants' Hospital. Coming from him, the assurance in his remarks is very satisfactory, and it made a great impression on all who heard them.

### ADDITIONAL HOSPITAL STAFFS FOR FRANCE.

The need for an increased number of hospital workers in France has led to the organising of a hospital staff there, with practitioners and specialists from Dublin under the direction of Lieut.-Colonel William Taylor, R.A.M.C., the President of the Royal College of Surgeons, Ireland. He has undertaken to be responsible for staffing the hospital for a period of twelve months, and his intention is to accompany the last of the groups of nine into which the Irish contingent will be divided. The hospital thus staffed will be taken over as a going concern, but it is only the *personnel* for which Colonel Taylor is responsible. Each of the groups referred to is expected to serve for three months, and to secure the assistance of more medical men the War Office is understood to be granting commissions for this period or for six months only. The medical officers are to be over forty years of age. Each group will comprise two physicians, three surgeons, an anæsthetist, an oculist, a pathologist, and a radiographer. If need be, it is believed that the hospital could be staffed by Irish nurses also. The first group represented Sir Patrick Dun's, Dr. Steevens', Mercer's, the Royal City of Dublin, the Royal Victoria, and the Adelaide and Richmond Hospitals. The next group will represent other institutions, and the movement, we learn, is assured of success.

### THE EFFECT OF "MODERATE RESTRICTION" OF ALCOHOL ON THE PUBLIC HEALTH.

In a very scholarly and succinct lecture at the Royal Institute of Public Health, Lord d'Abernon traced the influence on the mortality from alcoholism of the "moderate restrictions" upon the consumption of alcohol which have prevailed since early in the war. In the course of his lecture he pointed out that hitherto there has been no middle course regarded as feasible between the full freedom demanded by the publican and the compulsory total abstinence favoured by the teetotal crank. Circumstances, however, have conspired to make heavy drinking much less easy, without depriving the moderate drinker of his alcohol. In some striking Liverpool statistics, derived from official sources, Lord d'Abernon shows that in 1916 the deaths certified as due to alcoholism were but 54.6 per cent. of the numbers in 1913; deaths "connected with alcoholism" were 56.1 per cent. of the corresponding deaths; and deaths from cirrhosis of the liver were down to 76.9 per cent. The experience of the last two years, he concludes, has definitely established two facts of very great importance: it has shown that an enormous amount of preventible injury has been done to the community by the absence of fitting regulation of the drink traffic; and it has shown, also, that, contrary to the current dictum, it is possible in a very considerable degree to diminish intemperance by legislative and administrative action."

### WAR CHARITIES TO BENEFIT BY LOTTERIES.

A method of raising funds for charities that would have occasioned much surprise and opposition a few years ago is being carried out in Glasgow. For some time collecting-boxes have been placed on Glasgow tramcars, and by these means various charities have benefited. The general manager of the tramway system has now originated a "prize-drawing" system. Conductors are supplied with special numbered tickets, resembling tram tickets, which are sold for a penny each, and prizes amounting to over £200 a week are distributed to those persons who have winning numbers. To encourage conductors to push the tickets, a prize is also given to the conductor who has sold the winning ticket. The prizes are War Savings Certificates chiefly, and the interest in the scheme is proved by the fact that last month £5,732 was realised, and of this £4,300 has been allocated amongst war charities. Each ticket is printed in duplicate, one set being sold, the other being placed in five boxes containing 100,000 each. Ten prize tickets are then drawn from each box. After the extreme caution shown by members of Parliament in the matter of lottery bonds, a mild form of speculation as compared with the tramway scheme, it is amusing to contemplate that it is a Scottish burgh that is the first to institute what is unquestionably a money lottery pure and simple.

### AN INSTITUTIONAL WEDDING GIFT.

Sir William James Thomas—whose numerous benefactions to hospitals and whose magnificent service as treasurer of the Welsh Hospital at Netley have laid the Principality under a great debt to him—along with Lady Thomas was the recipient of an interesting wedding present from the board of management of the King Edward VII. Hospital, Cardiff, last week. The gift, a silver epergne, inscribed "in appreciation of an association with the hospital in which each co-operated, and thus most materially helped its efficiency and extended its resources," was handed to Sir William and Lady Thomas by Major-General H. H. Lee (chairman of the board), who remarked that, as assistant matron, Lady Thomas had served the institution strenuously and well, while Sir William for thirteen years had been one of its best friends. In responding, Sir W. J. Thomas paid a tribute to the splendid work of the hospital, adding that he had as his better half one who had done a great deal to relieve suffering. He was sure they would have a happy life, he said, because they had made up their minds to do all they could to make others happy.

### QUINN'S BUILDINGS, WATERLOO ROAD.

In the issue of The Hospital for May 5, 1917, on page 84, the insanitary state of Quinn's Buildings in the Waterloo Road was mentioned; and the statement of a South London doctor, when giving evidence at an inquest on a child who died there was quoted to the effect that this property belongs to the Ecclesiastical Commissioners. The Secretary to the Commission writes to inform us that these buildings do not belong and never have belonged to the Ecclesiastical Commission. But for the fact that a reputable professional man, giving evidence on oath, committed himself to the contrary effect, it seemed scarcely credible that the slum property in question could be so mal-administered by a public body of high standing such as the Ecclesiastical Commission, and it gives us great pleasure to learn that the facts are as stated by the Secretary, and to inform all concerned that this is so.

### THE KING EDWARD VII. HOSPITAL, WINDSOR.

Cardiff is not, of course, the only town which possesses a hospital named after his late Majesty King Edward. At Windsor, appropriately enough, there is also a King Edward VII. Hospital, and here, too, as at Cardiff, financial difficulties seem to be in a fair way to be solved by the generosity and patriotism of local residents. There is a debt at Windsor of £1,925, towards the extinction of which King George V. has sent a donation of £200. A lady has offered to provide a further £1,000 provided the balance is subscribed by the end of this month. Already there has been a good response to this challenge, and the debt is now narrowed down to not much over £300. If this can be secured before the beginning of June, the hospital will obtain the conditional £1,000 just mentioned, and will be freed from a load of debt which has pressed heavily upon it. We trust and believe that the local residents of all classes will unite to provide this comparatively small sum, which should be well within the limits of attainment.

### WAR BONUS FOR INFIRMARY CHAPLAINS.

THE Kensington Board of Guardians have decided to apply to the Local Government Board to sanction an addition of £30 per annum to the salary of the Rev. A. Lombardini, chaplain of the infirmary and institution. Mr. Lombardini was appointed in September 1912 at a stipend of £250, with an additional £25 per annum for burial services. In applying for an extra grant or bonus the chaplain pointed out that not only had his ordinary and travelling expenses necessarily increased, but that numbers of cases from Fulham and Hammersmith have been admitted, which have increased his work. The number of these cases at the beginning of the month was 527. His request received the support of the institution's committee, which praised the way in which the additional work had been performed. The chaplain is surely as much entitled to a war bonus as any other official, and the only condition attached to the recommendation is that the grant, if sanctioned, shall continue so long as the additional inmates from outside parishes are received.

### ANOTHER CANCER WING IN LONDON.

THE number of general hospitals with special wards or blocks for the treatment of cases of cancer is increasing, and the latest addition to their number is the West London Hospital. An important bequest for this purpose has been received under the will of the late Mrs. Hull Martin, whereby a sum of £15,000 will be spent on the creation of a special cancer wing. The problem, of course, is one not only of beds, but of equipment and maintenance, and the proposed wing will doubtless become a self-contained unit. If there is no endowment its maintenance may cause some difficulty, but cancer seems still a word to conjure with, and public generosity has been stimulated by the war in a way which we believe will outlast the time of trial that evoked it.

### THE CLAYTON HOSPITAL AND VENEREAL DISEASES.

THANKS to the facilities offered at the clinic for venereal diseases at the Leeds General Hospital, the Wakefield City Council has been relieved of a difficulty into which it was thrown by the refusal of the Clayton Hospital, Wakefield, to treat these cases. It is felt at Wakefield that the governors of the Clayton Hospital had made up their minds before meeting the deputation from the City Council. Even the Mayor, in a guarded speech, declared that though he did not believe the governors had intended to be discourteous, he considered that they had been badly advised. It would be interesting to know on what grounds the Clayton Hospital came to a decision which places it outside a movement which the voluntary hospitals have as a whole eagerly combined to help.

### GIANT AT A HOSPITAL.

ON Tuesday last a giant walked into the out-patient department of the National Hospital for the Paralysed and Epileptic, and after he had been seen by the out-patient physician was admitted as an urgent case. This patient, who measures 8 feet 2 inches in height, now occupies two beds, and we are told by the hospital authorities that he is the first giant admitted this year. It is possible that Lord Devonport will have to be consulted about the patient, as, owing to his appetite, the average bread consumption of the ward has increased. The plate of bread and butter intended for the whole ward was inadvertently left on the giant's locker while sister went round pouring out the breakfast tea; the patient quite innocently thought it was his allowance, and ate most of the ward supply before the mistake was noticed.

### THE NATIONAL BABY WEEK.

THE Council which is "running" the National Baby Week (July 1 to 7) is taking Time well by the forelock and leaving nothing to chance. Already a number of telling pamphlets and leaflets are ready, for the use of speakers, lecturers, clergy, and others of the instructed upon whom the task will devolve of leavening the lump of British ignorance about babyhood. Our readers are probably aware that Mr. Lloyd George, the Prime Minister, is the president of this Council; and that Lord Rhondda, the President of the Local Government Board, is the chairman. The offices of the Council are at 6 Holles Street, Oxford Street, W., where the secretary, Miss Elliott, will answer all inquiries and welcome all offers of assistance. The problem of conserving the precious lives now wasted through maternal ignorance and indifference, poverty, drink, bad housing, overcrowding, and similar avoidable evils is one whose solution is vital to our existence as a nation; and the National Baby Week is a courageous attempt to solve at least a portion of it.

### THE EXPOSURE OF FOOD.

IN Parliament a few days ago Captain Bathurst stated that it was proposed to publish shortly an Order making the waste of any kind of food a punishable offence. By what machinery such a reform could be effected it is not easy to see. A daily house-to-house inspection, or rather inquisition, might conceivably be instituted, and the vast army of officials required would be in the best Governmental tradition. Conceivably, a head inspectorship of dustbins might afford an agreeable sinecure for one who by judicious wirepulling had deserved well of his party. But a more pregnant reform which does not seem as yet to have occurred to the Food Controller is the prohibition of the public display of all kinds of foodstuff. The wastage of food entailed by the barbaric displays of the butcher, the fishmonger, and the greengrocer is enormous, and will increase tenfold with the approaching summer. Apart from æsthetic reasons—the sight of an eviscerated ox or sheep does not seem to disturb the timid matron who would flee in terror from a live mouse!—the exposure of meat to contamination by countless flies, and the dust from a busy highway laden with bacteria and the dry excreta of animals cannot fail to have a most injurious effect on those who consume it. Fruit goes rotten and fish and meat become tainted long before they need. Surely the time for shop-window displays has gone by. Their only effect is to make people eat more, the very thing we wish to avoid.

### WHAT LANCASHIRE EATS TO-DAY.

Hospitals which are finding it a daily increasing difficulty to obtain potatoes, not to mention anxieties regarding sugar, will learn with envy from Dr. Wheeler Hart, the Lancashire director of the British Red Cross Society, that every auxiliary hospital in East Lancashire has been provided with supplies of potatoes sufficient to carry them through until the end of June. At one time the hospitals were also practically without sugar, but Dr. Hart got over that difficulty by registering himself as a sugar agent. In regard to sugar for infants, it is interesting to note that an arrangement has been entered into with the Sugar Commission by Dr. Niven, the Manchester M.O.H., for a guaranteed supply, which will be sold, through the schools for mothers, to necessitous cases in Manchester, at cost price.

### BEWARE OF RHUBARB LEAVES.

The recent case reported in the newspapers in which a death occurred as the result of eating rhubarb leaves as a " green " vegetable was curious in that the recipe appeared to be recommended by a body which had used it for a long period without any untoward result. Yet, we believe, disastrous results of more or less gravity have not been confined by any means to the unfortunate person on whom the inquest was held. The scarcity of vegetables and the campaign in favour of food economy is leading to the advertisement of a lengthy list of substitutes, and patriotic persons, under this double stimulus, are apt to consume food which they had better avoid. Almost simultaneously with the case in question we heard of a man and his wife who were made severely ill by eating rhubarb leaves in this way. It is very probable that they saw the same announcement as was seen by the unfortunate person who died. In the cases which came within our knowledge recoveries were happily made; but the moral is that caution is much needed before food " substitutes " are consumed by all and sundry without any apprehension of the risks which may attend a meal mainly consisting of them.

### HOSPITAL "GAZETTES" AND THE SUPPLY OF PAPER.

The shortage of paper and its rise in price have induced at least one hospital *Gazette* to announce that its publication will be discontinued. This, unfortunately, is the *Craigleith Hospital Chronicle*, one of the best of the war hospital newspapers, which has done as much as any to maintain a high standard of appearance and a high level of interest in its contents. Only two more regular monthly numbers are promised, but it is hoped that the editor may be able, like the parish priest, to issue an occasional paper from time to time. Among the more prosaic of the *Chronicle's* achievements has been the enlargement of the hospital's billiard-room, the entire cost of which has been met out of its profits. The *Gazette* of the 3rd London General Hospital is not one of those which at present confesses to a shortage of paper. It is still as prolific as ever of illustrations. The May number confines itself mainly to sketches in which the search after humour is somewhat continuously strained, but we hope the old ideal of a more varied and less hackneyed appeal will not be abandoned. Pte. Ernest Collings provides a frontispiece entitled " Sleep." The photographs include portraits of the contributors who have helped to make the *Gazette* a success, and the most successful drawings include those of Sergeant George Coates, entitled " Melody Makers " and the " Electrophone." In the articles of the May number there is nothing which calls for special mention.

### THIS WEEK'S DRUG MARKET.

In view of prevailing high prices and of the abnormal conditions ruling in the market, it is not surprising that business is quiet and that transactions are confined to a small scale. Following on the Army Council's Order with regard to certain drugs, the Government have taken over a fair quantity of quinine, leaving holders at liberty to sell their remaining stocks in the ordinary way of business; quinine can now be bought at a price not very substantially higher than that which was ruling immediately before the publication of the Army Council's Order. With regard to phenacetin there have been no further developments, and dealings are restricted to small quantities; the price is very high. Small transactions have taken place in formaldehyde at higher rates than those that were being paid before the intervention of the Government. Clove oil is much dearer, and still higher prices are expected. Sodium salicylate still has an upward tendency in price. Sulphonal is dearer. Business has been done in milk sugar at a very high figure. Reports from the Norwegian fishing grounds are to the effect that the progress of the cod fishing is not good and the yield of cod-liver oil is below the average; none of the new season's oil appears to be offered in this market, and the price for available supplies of last season's oil is likely to remain high.

### TO OUR READERS.

Contributions are specially invited from any of our readers to these columns. They should deal with topical subjects and news. They must be authenticated for the information of the Editor only. The minimum payment if published is 5s. There is no hard-and-fast rule as to space, but notes of about twenty lines in length are preferred.

